# Experimental evidence of the genetic hypothesis on the etiology of bicuspid aortic valve aortopathy in the hamster model

**DOI:** 10.3389/fcvm.2022.928362

**Published:** 2022-08-08

**Authors:** María Teresa Soto-Navarrete, Bárbara Pozo-Vilumbrales, Miguel Ángel López-Unzu, Carmen Rueda-Martínez, M. Carmen Fernández, Ana Carmen Durán, Francisco Javier Pavón-Morón, Jorge Rodríguez-Capitán, Borja Fernández

**Affiliations:** ^1^Departamento de Biología Animal, Facultad de Ciencias, Universidad de Málaga, Málaga, Spain; ^2^Instituto de Investigaciones Biomédicas de Málaga y Plataforma en Nanomedicina, Universidad de Málaga, Málaga, Spain; ^3^Spanish National Centre for Cardiovascular Research, Madrid, Spain; ^4^Departamento de Anatomía Humana, Medicina Legal e Historia de la Medicina, Facultad de Medicina, Universidad de Málaga, Málaga, Spain; ^5^Centro de Investigación Biomédica en Red Enfermedades Cardiovasculares (CIBERCV), Málaga, Spain; ^6^Unidad de Gestión Clínica del Corazón, Hospital Universitario Virgen de la Victoria, Málaga, Spain

**Keywords:** bicuspid aortic valve (BAV), aortic dilatation, pathophysiology, etiology, animal model, hamster

## Abstract

Bicuspid aortopathy occurs in approximately 50% of patients with bicuspid aortic valve (BAV), the most prevalent congenital cardiac malformation. Although different molecular players and etiological factors (genetic and hemodynamic) have been suggested to be involved in aortopathy predisposition and progression, clear etiophysiopathological mechanisms of disease are still missing. The isogenic (genetically uniform) hamster (T) strain shows 40% incidence of BAV, but aortic dilatations have not been detected in this model. We have performed comparative anatomical, histological and molecular analyses of the ascending aorta of animals with tricuspid aortic valve (TAV) and BAV from the T strain (TTAV and TBAV, respectively) and with TAV from a control strain (HTAV). Aortic diameter, smooth muscle apoptosis, elastic waviness, and *Tgf*-β and *Fbn-2* expression were significantly increased in T strain animals, regardless of the valve morphology. Strain and aortic valve morphology did not affect *Mmp-9* expression, whereas *Mmp-2* transcripts were reduced in BAV animals. eNOS protein amount decreased in both TBAV and TTAV compared to HTAV animals. Thus, histomorphological and molecular alterations of the ascending aorta appear in a genetically uniform spontaneous hamster model irrespective of the aortic valve morphology. This is a direct experimental evidence supporting the genetic association between BAV and aortic dilatation. This model may represent a population of patients with predisposition to BAV aortopathy, in which increased expression of *Tgf*-β and *Fbn-2* alters elastic lamellae structure and induces cell apoptosis mediated by eNOS. Patients either with TAV or BAV with the same genetic defect may show the same risk to develop bicuspid aortopathy.

## Introduction

Bicuspid aortic valve (BAV) is the most frequent congenital cardiac malformation, with an incidence of 1–2% in the general population. Approximately 50% of affected individuals develop dilatation of the proximal aorta (AD), a condition named bicuspid aortopathy. Affected patients are at risk of dissection and rupture of the artery with fatal consequences (1).

Cystic medial degeneration, characterized by elastic fiber fragmentation and smooth muscle cell (SMC) apoptosis, is the histopathological substratum of BAV aortopathy (2). Defective deposition of Fibrillin-1 (FBN-1) in the media has been proposed as an early trigger, causing SMC detachment and death (3–5). Fibrillin-2 (FBN-2) seems also involved, as its transcription is up-regulated in some patients with BAV and AD (5). Alterations of FBN content and/or composition in the aortic media may cause deregulation of the *Transforming growth factor-β* (*Tgf-β*) pathway, as it occurs in other syndromic aortopathies. This would alter the vascular matrix homeostasis through over-activation of metalloproteases (MMPs), leading to aortic wall degeneration (6–8). As to the effect of hemodynamics in AD progression, Endothelial nitric oxide synthase (eNOS) has been suggested to be a central molecule. Its expression is shear-stress dependent and changes significantly in the convexity and concavity of the aneurysmatic aorta according to the aortic valve morphology (9, 10). Despite significant advance in our knowledge on AD pathobiology, we still do not have a clear picture of the different cellular and molecular mechanisms of the disease.

During the last decade, there has been an intense debate on the etiology of bicuspid aortopathy (genetic, i.e., structural vs. environmental, i.e., hemodynamic). The hemodynamic hypothesis states that BAV morphology causes alterations of the blood jet stream, leading to deterioration of the aortic wall histological structure over time. The genetic hypothesis proposes that the genetic trigger of BAV formation during embryonic development leads also to a congenital structural defect of the ascending aorta, which predisposes to aortic wall degeneration (6, 11–15). However, direct empirical evidence supporting the genetic hypothesis is still missing.

Probably the greatest current difficulty in verifying the influence of genetic factors on bicuspid aortopathy results from the fact that the population of affected patients is genetically heterogeneous, with different clinical presentations associated with different disease etiologies (4, 15–18). Different mutant murine models have been developed in order to uncover the etiology of BAV. These models have substantially improved our knowledge on BAV embryonic development [reviewed in Ref. (19)], but only a few of the genes modified in the mutant models have been shown to cause BAV disease in a minority of affected patients (20–23). In addition, most of the mutant mice with BAV show a combination of cardiac and extracardiac congenital malformations, thus probably representing syndromic BAV instead of the most common isolated BAV. Bicuspid aortopathy similar to that occurring in BAV patients have not been described in these models. It is currently recognized the necessity of developing animal models that properly mimics human BAV aortopathy pathogenesis, in order to understand the etiopathogenesis of the disease and to undergo preclinical research (24).

Until now, only one spontaneous animal model of isolated non-syndromic BAV has been described. It consists of an inbred (i.e., isogenic) strain (T strain) of Syrian hamster (*Mesocricetus auratus*) with a relatively high (∼40%) incidence of BAV (25–29). The anatomy and inheritance of BAV in the hamster model are similar to those in patients (25, 27). The penetrance of the trait is incomplete, so that both BAVs and tricuspid aortic valves (TAVs) can be found in genetically identical hamsters and patients (11, 27, 30–32). Aortic aneurysm or structural alterations of the aortic wall have never been described in the hamster model.

In the present paper, we have studied the ascending aorta of animals from the hamster model of BAV, looking for possible structural alterations that could validate the hamster T strain as an animal model for bicuspid aortopathy predisposition. With this aim, we examined the anatomy and histomorphology of the ascending aorta, and the expression of several genes and proteins known to be involved in AD pathogenesis (*Fbn-1*, *Fbn-2*, *Tgf*-β, *Mmp-2*, *Mmp-9*, and eNOS). These studies were performed comparing animals from the T strain (inbred), including genetically homogeneous animals with BAV and TAV, and TAV animals from a control outbred line with a null incidence of BAV. Thus, signs of ascending aorta alterations in BAV vs. TAV hamsters, irrespective of their strain origin, would favor hemodynamic alterations caused by the valve anatomy as the leading cause of the arterial defects. Conversely, aortic alterations in hamsters of the T vs. the control strain, irrespective of their valve anatomy, would point to genetic factors as the main etiological origin.

## Materials and methods

### Animals

The hamster model of BAV consists of an inbred strain of Syrian hamsters (*M. auratus*) with roughly 40% incidence of spontaneously appearing BAV type A [R/L fusion type, including fused BAV, 2 sinus BAV and partial fusion BAV, according to Ref. (1)]. The characteristics of this unique inbred strain (from now T or affected strain) have been previously reported (24–28). For comparative purposes we selected an outbred colony (RjHan:AURA; Janvier, France; from now H or control strain) previously used as a control for T strain (29, 33).

Animals were handled in accordance with the European guidelines (Directive 2010/63/EU) and Spanish regulations (R.D. 53/2013; B.O.E. 01.02.2013) for the protection of experimental animals. The protocol was approved by the Ethics Committee of Animal Experiments of the University of Málaga (CEUMA; Ethics authorization number: 25/08/2021/118).

The animals were euthanized by inhalation of CO_2_, and their body length was measured. Heart and thoracic arteries were removed and washed with 0.1 M phosphate buffered saline (PBS; pH 7.3). The ascending aorta, from the sinotubular junction to the branching of the brachiocephalic artery was excised and processed for anatomical and histomorphological analyses or mRNA extraction. Aortas processed for histomorphology were dissected longitudinally to separate convexity and concavity. The heart was further processed for evaluation of the aortic valve morphology.

For the anatomical and histomorphological studies, a total of 45 animals (31 male, 14 female) were grouped according to the strain (T or H) and the aortic valve morphology (BAV or TAV): H-TAV (*n* = 18); T-TAV (*n* = 16); T-BAV (*n* = 11). These 45 specimens were old animals (300–500 days old).

For the mRNA quantification studies, a total of 107 animals (48 male, 59 female) were grouped according to the strain, the aortic valve morphology and the age (adult: 180–240 days old; old: 300–440 days old): H-TAV-adult (*n* = 15); H-TAV-old (*n* = 15); T-TAV-adult (*n* = 21); T-TAV-old (*n* = 24); T-BAV-adult (*n* = 15); and T-BAV-old (*n* = 17).

### Anatomy

The condition of the aortic valve was assessed by means of stereomicroscopy. Then, the aortic valve was removed and processed for scanning electron microscopy as previously described (26). A small (∼1 mm long) segment of the proximal ascending aorta was dissected just after extraction, placed on a scale under the binocular microscope, and the relative diameter (aortic diameter to body length ratio) was calculated for each specimen.

### Histomorphology, apoptosis, and immunoperoxidase

The concavity and convexity of the ascending aorta were separately fixed by immersion in 4% paraformaldehyde in PBS overnight, dehydrated in graded ethanol and embedded in paraffin (Histosec; Merck KgaA, Darmstadt, Germany). Nine μm serial transverse sections of the middle portion of each segment were used to quantify elastic lamina waviness index, SMC apoptosis and eNOS expression.

The waviness index is a measure of the degree of undulation of the elastic lamellae, defined as the ratio of the lamellar length to the straight-line distance between two points (34, 35). A total of 15 animals were analyzed: H-TAV (*n* = 5); T-TAV (*n* = 5); T-BAV (*n* = 5). It was measured in microphotographs of sections of the convex and concave regions of the aorta using elastic lamina autofluorescence (Supplementary Figure 1). The length of each lamellae was measured in three randomly selected optical fields (×200) per section of the aortic convexity and concavity. At least three non-consecutive sections per animal were used.

Smooth muscle cell apoptosis was detected by means of the terminal deoxynucleotidyl transferase-mediated deoxyuridine triphosphate nick end labeling (TUNEL) assay in a total of 15 animals: H-TAV (*n* = 5); T-TAV (*n* = 5); T-BAV (*n* = 5). A commercial kit (Roche, Switzerland) was employed following the manufacturer’s instructions. Omission of the transferase enzyme was used as a negative control. After the TUNEL assay, the sections were stained with 4′,6-diamidino-2-phenylindole dihydrochloride (DAPI, Sigma-Aldrich, United States) diluted 1:2,000 in PBS to verify the nuclear location of the TUNEL signals. The sections were observed and photographed with a Leica SP8 confocal microscope. The number of DAPI^+^ and TUNEL^+^ cells were counted in three randomly selected optical fields (×400) per section of the aortic region convexity and concavity, and the incidence of apoptotic cells was calculated. At least three non-consecutive sections per animal were used.

Expression of eNOS was studied by immunoperoxidase. It was performed as previously described (36) in a total of 15 specimens: H-TAV (*n* = 5); T-TAV (*n* = 5); T-BAV (*n* = 5). eNOS polyclonal antibody (Ref.: N3893, Sigma-Aldrich, United States) diluted 1:50 was used. Secondary antibody was biotin-conjugated anti-rabbit IgG (Sigma-Aldrich, United States) diluted 1:1,000. Omission of the first antibody served as a negative control. The sections were photographed using a Leica DMSL microscope equipped with a Leica DFC500 camera. The area of the media occupied by eNOS^+^ cells was quantified in microphotographs using the open-source software FIJI. The aortic media was delineated manually, and the complete area was measured. A threshold (min. 150; max. 190 in a gray scale) was stablished to delimit the positive signals and avoid background. The ratio eNOS^+^ area/total area of the aortic media was calculated. The quantification was performed in three randomly selected optical fields (×200) per section of the convexity and concavity. At least three non-consecutive sections per specimen were used.

### Quantitative real time polymerase chain reaction

The ascending aorta was cryopreserved in liquid nitrogen and stored at −80°C for subsequent RNA extraction. Due to the small size of the hamster aorta, two or three aortic specimens were included in each sample. Thus, a total of 38 samples were used: H-TAV-adult (*n* = 5); H-TAV-old (*n* = 7); T-TAV-adult (*n* = 7); T-TAV-old (*n* = 8); T-BAV-adult (*n* = 5); T-BAV-old (*n* = 6).

RNA isolation, cDNA synthesis and qPCR analyses were performed as previously described (33), using the same samples. The sequence primers for *Mmp-9*, *Fbn-1*, and *Fbn-2* were designed by Primer3, whereas those for *Tgf*-β and *Mmp-2* were obtained from previously published papers (Supplementary Table 1). The mRNA expression of target genes was normalized against the reference gene *Cdkn1β* (5, 33). The amplification efficiency and correlation coefficient were calculated using LingRegPCR software (Supplementary Table 2). The ΔCt method was used for normalization and quantification of expression values.

### Statistical analysis

All graphics and data show the mean and the standard error of the mean. Differences between two groups were analyzed using the Student’s *t*-test and were considered statistically significant when *p* < 0.05. The statistical analyses were performed with the SPSS 27.0 software.

## Results

### Aortic valve morphology

**FIGURE 1:**
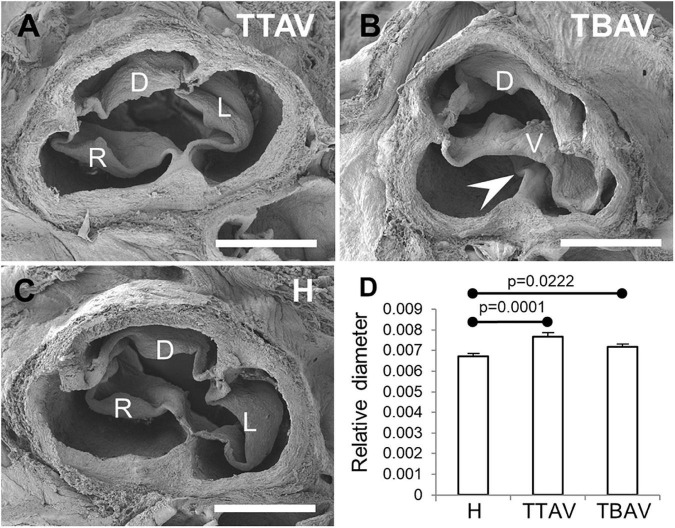
Scanning electron micrographies of TAVs **(A,C)** and a BAV **(B)** of hamsters from the affected **(A,B)** and the control **(C)** strains. Cranial views. D, dorsal (non-coronary) leaflet; L, left leaflet; R, right leaflet; V, ventral leaflet. Arrowhead: raphe. Scale bar: 500 μm. **(D)** Analysis of the relative diameter of the ascending aorta of hamsters grouped according to the strain and the valve morphology. H, control strain; TBAV, bicuspid aortic valve from the affected strain; TTAV, tricuspid aortic valve from the affected strain. Only significant *p*-values are detailed.

Figure 1 shows representative aortic valves of animals from the H and T strains. All animals (*n* = 43) from the H strain (Figure 1C) and 57 out of 99 (57.6%) from the T strain (Figure 1A) had TAVs. These were composed of three leaflets and three sinuses, right, left and dorsal (non-coronary). In the T strain, 42 out of 99 (42.4%) animals had BAVs (Figure 1B) composed of two leaflets and two sinuses with a dorsoventral (antero-posterior in humans) orientation [2-Sinus BAV, according to Ref. (1)]. In the middle of the ventral aortic sinus of most BAVs, a raphe could be distinguished, sometimes encroaching toward the ventral leaflet [Fused BAV, according to Ref. (1)].

### Aortic size

Observations under the stereomicroscope revealed no obvious aneurism in the ascending aorta or aortic root in any of the specimens studied. However, ascending aorta measurements revealed that animals from the T strain showed a significant increase in aortic diameter (mean aortic diameter: 1.038 mm) as compared with animals of the control strain (mean aortic diameter: 1.019 mm), either when considering the aortic valve morphology (Figure 1D) or not (Supplementary Figure 2).

### Histomorphology and apoptosis

**FIGURE 2:**
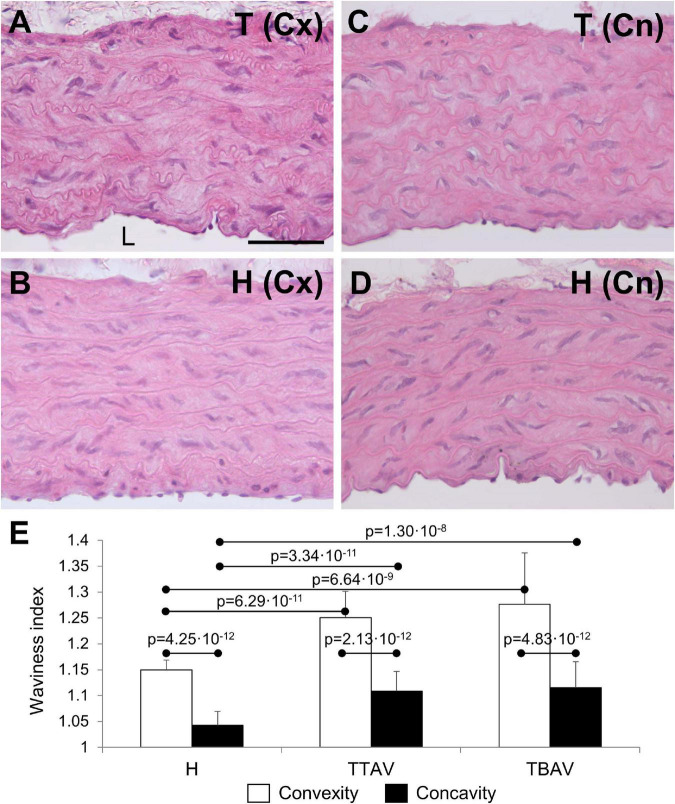
Transverse sections of the ascending aorta convexity (Cx; **A,B**) and concavity (Cn; **C,D**) of hamsters from the affected **(A,C)** and the control **(B,D)** strains, stained with hematoxylin and eosin. Scale bar: 100 μm. **(E)** Quantification of the waviness index in the aortic convexity and concavity of animals from the control strain (H) and from the affected strain with TAV (TTAV) and BAV (TBAV). Only significant *p*-values are detailed.

The ascending aorta of animals from both strains showed the normal histological structure of a rodent artery (Figures 2A–D). The aortic wall was composed of nine to eleven lamellae. The undulating sheets of elastic fibers were continuous, without evident disruptions. However, the undulations in animals of the T strain were apparently more pronounced. Waviness index quantification showed significantly more undulated elastic lamina in the convexity than in the concavity of all animals (Figure 2E). Hamsters from the T strain, either with BAV or TAV showed a significantly increased waviness index compared with control hamsters in both the aortic convexity and concavity (Figure 2E). When animals were grouped according to the strain or the valve morphology, differences remained significant (Supplementary Figure 3).

**FIGURE 3:**
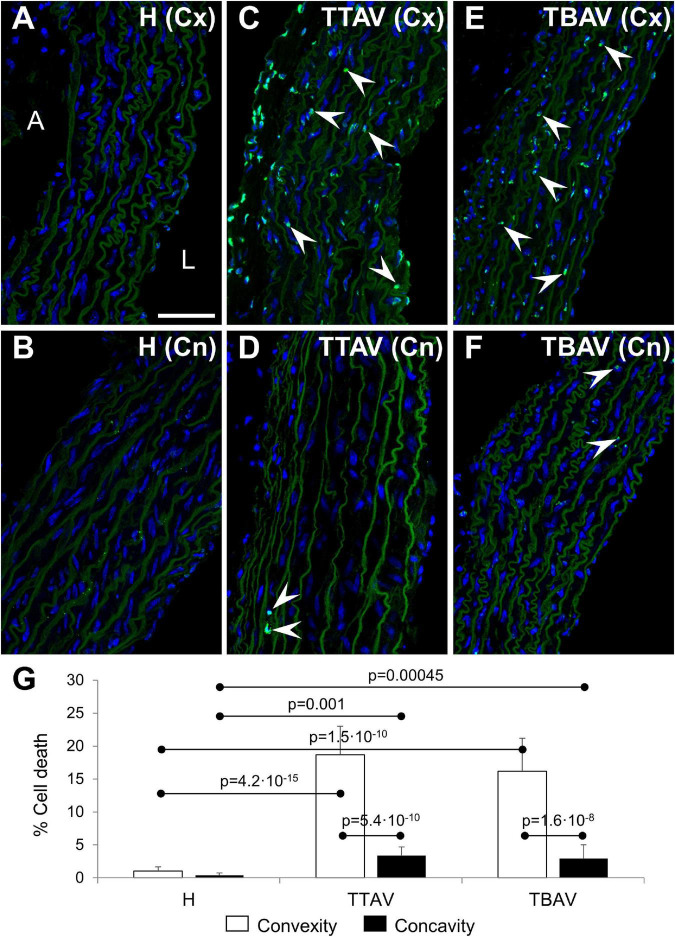
Confocal images of the convexity (Cx; **A,C,E**) and concavity (Cn; **B,D,F**) of the ascending aorta of hamsters from the control **(A,B)** and affected **(C–F)** strains. TUNEL fluorescence (green) demonstrated abundant apoptotic cells (arrowheads) in the media of the aortic convexity of animals of the affected strain both with TAV **(C)** and BAV **(E)**. DAPI (blue) stained nuclei. No TUNEL signal was detected in aortas from control animals **(A,B)** and in the negative control (not shown), and only a few signals were found in the concavity of animals of the affected strain **(D,F)**. Note that the elastic sheets show some autofluorescence. A, adventitia; L, lumen. Scale bar: 50 μm. **(G)** Quantification of the percentage of TUNEL^+^ cells revealed significant differences between convexity and concavity, and between the affected and control strains, irrespective of aortic valve morphology. H, control strain; TBAV, bicuspid aortic valve from the affected strain; TTAV, tricuspid aortic valve from the affected strain. Only significant *p*-values are detailed.

The TUNEL assay revealed abundant apoptosis in the aortic media of animals of the T strain (Figures 3C,E), whereas it was almost absent in control animals (Figures 3A,B). Apoptosis quantification showed almost 10 times more TUNEL^+^ cells in the aortic convexity than in the concavity of affected animals (Figure 3 and Supplementary Figure 4A). Differences and significances were even larger when comparing animals from the control and the T strain (Figure 3G and Supplementary Figure 4A). However, no significant difference was found between animals with BAV and TAV, either in the aortic convexity or the concavity (Figure 3G and Supplementary Figure 4B).

### Endothelial nitric oxide synthase immunohistochemistry

**FIGURE 4:**
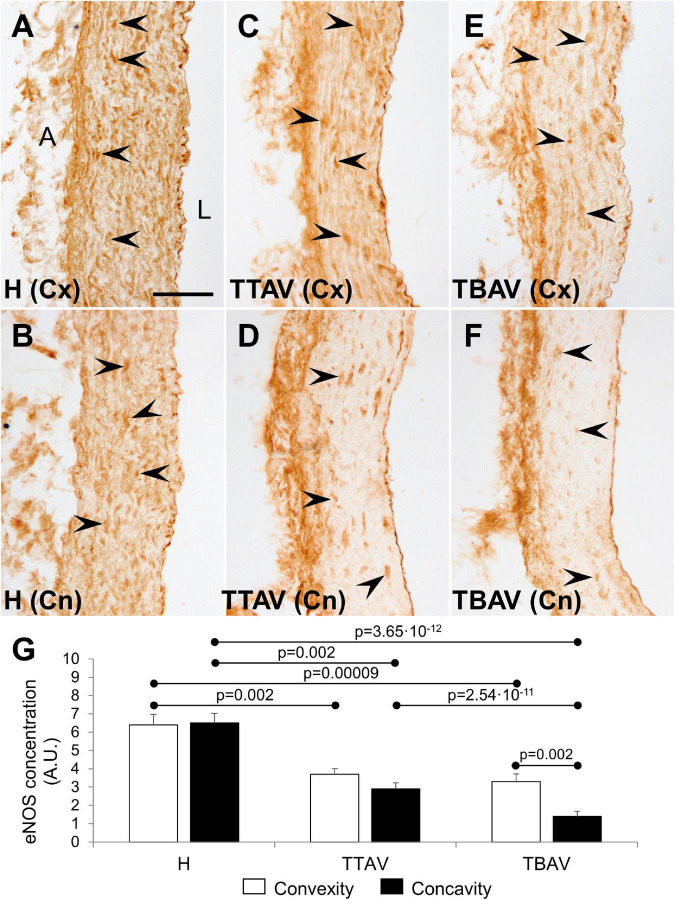
Endothelial nitric oxide synthase immunoperoxidase in the convexity (Cx; **A,C,E**) and concavity (Cn; **B,D,F**) of the ascending aorta of hamsters from the control **(A,B)** and the affected **(C–F)** strains. All specimens showed strong immunoreactivity in the endothelium, close to the lumen (L), and the adventitia (A). Medial eNOS^+^ smooth muscle cells (arrowheads) were abundant and homogeneously distributed in animals from the control strain **(A,B)**, but they were scarce and scattered in animals of the T strain **(C–F)**, particularly at the aortic concavity **(D,F)**. No signal was detected in the negative control (not shown). Scale bar: 50 μm. **(G)** Quantification of the aortic media area occupied by eNOS^+^ cells. Expression was reduced almost by halve in both the convexity and the concavity of animals of the T strain. Convexity and concavity showed similar eNOS expression except in animals with BAV, in which the reduction in the concavity was even more pronounced. H, control strain; TBAV, bicuspid aortic valve from the affected strain; TTAV, tricuspid aortic valve from the affected strain. Only significant *p*-values are detailed.

Immunostaining with anti-eNOS antibody showed a large number of positive cells in the aortic media of animals from the control strain (Figures 4A,B). However, the signal was lower and scarce in animals of the T strain either with TAV (Figures 4C,D) and BAV (Figures 4E,F). Endothelial cells were equally positive in both strains. Quantification of the aortic media area occupied by eNOS^+^ cells revealed reduction of eNOS expression both at the convexity and concavity of animals of the T strain, being more pronounced in the concavity of BAV animals (Figure 4G and Supplementary Figure 5).

### Quantitative real time polymerase chain reaction

**FIGURE 5:**
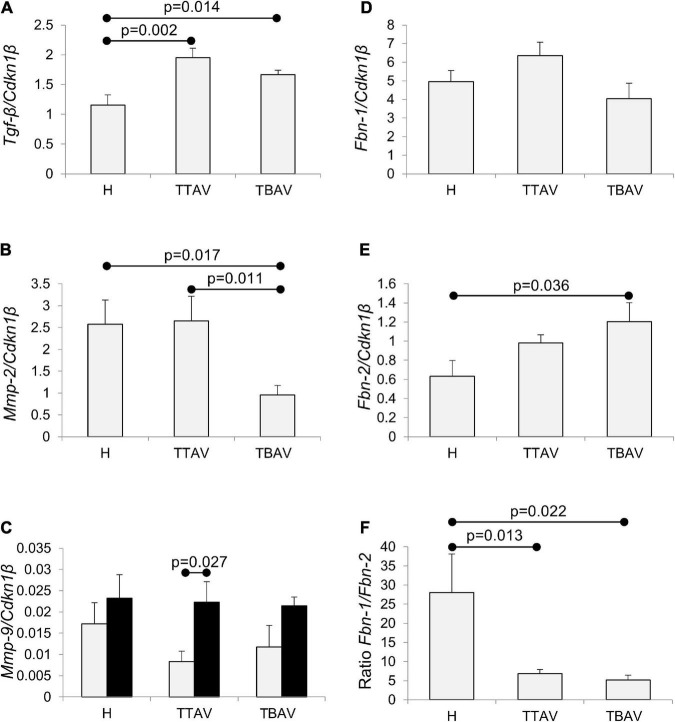
Analysis of *Tgf-*β, *Mmp-2*, *Mmp-9*, *Fbn-1*, and *Fbn-2* mRNA expression in the ascending aorta of hamsters grouped according to the strain and the valve morphology. *Tgf-*β expression **(A)** was significantly higher in animals of the affected strain irrespective of aortic valve morphology. *Mmp-2* expression **(B)** was significantly reduced in animals with BAV from the affected strain. *Mmp-9* expression **(C)** increased with age, although significantly only for T-TAV animals, but did not change significantly with the strain and the valve morphology. *Fbn-1* expression **(D)** did not significantly change in animals of the affected strain, both with TAV and BAV. *Fbn-2* expression **(E)** was higher in animals of the affected strain, although differences were significant only for animals with BAV. *Fbn-1*/*Fbn-2* expression ratio **(F)** was over fivefold reduced in animals from the T strain, both with TAV and BAV. The expressions were normalized against the reference gene *Cdkn1*β. H, control strain; TBAV, bicuspid aortic valve from the affected strain; TTAV, tricuspid aortic valve from the affected strain. Only significant *p*-values are detailed.

The expression levels of the five genes studied in the ascending aorta of adult and old animals belonging to the three animal groups established (H, T-TAV, and T-BAV) are shown in Figure 5 and Supplementary Figure 6. For the genes *Tgf*-β, *Mmp-2*, *Fbn-1*, and *Fbn-2*, no significant differences between adult and old animals were detected (Supplementary Figure 6), thus, data were grouped.

The levels of expression of *Tgf*-β were around 50% significantly higher in the aorta of animals from the T strain either with TAV or BAV (Figure 5A and Supplementary Figure 7A). However, no significant differences were obtained when comparing T-TAV with T-BAV animals (Figure 5A).

The expression levels of *Mmp-2* were significantly higher in the aorta of animals with TAV vs. BAV, either considering all animals with TAV (Supplementary Figure 8) or considering the control and affected strains separately (Figure 5B). However, no significant differences were obtained between strains (Supplementary Figure 8).

*Mmp-9* expression increased with age in all the groups compared (Figure 5C and Supplementary Figure 9), although differences were significant only for animals with TAV from the T strain. No differences were obtained between animals from different strains or with different valve morphologies.

While *Fbn-1* expression did not change significantly in any of the groups examined (Figure 5D and Supplementary Figures 10A,D), the expression of *Fbn-2* was 1.71-fold significantly higher in the aorta of animals from the T strain (Supplementary Figure 10B), specifically in animals with BAV (Figure 5E). Consequently, the ratio of expression of both isoforms (*Fbn-1*/*Fbn-2*) was very much reduced in the aorta of animals from the T strain, both with TAV and BAV (Figure 5F).

## Discussion

The T strain of Syrian hamsters constitutes a well-established spontaneous animal model of non-syndromic congenital BAV (1, 3, 6, 11, 24–28, 32). In the animal cohort studied here, antero-posterior BAV, the most common human BAV phenotype (1, 12), was found in 42.4% of the specimens, whereas conspicuous dilatations of the ascending aorta were not observed. However, we found that, compared with a control strain, hamsters of the T strain either with BAV or TAV present alterations of the histological structure of the aorta, as well as of the expression profile of genes known to be relevant for AD pathogenesis. The histomorphological alterations consist of SMC apoptosis and wavier elastic lamellae, especially in the convexity of the ascending aorta, and increased aortic diameter. The molecular alterations consist of increased *Tgf-β* and *Fbn-2* expression and reduced eNOS abundance. All these alterations, both morphological and molecular, occurred in animals of the T strain, irrespective of the aortic valve morphology. Given that human BAV aortopathy is characterized by regional medial degeneration (higher affection in convexity), including altered elastic fibers and increased apoptosis, as well as deregulation of specific molecular pathways such as eNOS, TGF-β, and FBN (1–4, 6, 9, 37), we propose that the T strain of hamsters is an animal model of BAV aortopathy predisposition. The alterations detected in the aorta of the hamster model may represent an early stage in the pathogenesis of AD associated with BAV. The high incidence of SMC apoptosis, especially in the aortic convexity, is particularly revealing, given the specificity of this alteration for BAV aortopathy in patients. The lack of progression of aortic wall alterations to aortopathy in this model may have different causes, including the short average life expectancy of the Syrian hamster, or possible inter-specific differences, including biomechanical differences with humans due to the very small size of the hamster aorta. Although BAV is inherited in this model (25–27), we still do not know the genetic triggers involved. Omic strategies applied to the three experimental groups studied here (TTAV, TBAV, and H) may help elucidating these triggers. However, the fact that this model appeared spontaneously, and both anatomy and inheritance are similar to those in affected patients, point to a probably high degree of extrapolation from the T strain to human, compared to genetically engineered animal models. Indeed, while genetic modifications in 28 genes cause BAV and/or aortopathy in mouse models (24, 29), only variants in 6 of these genes (*NOTCH1*, *GATA5*, *GATA6*, *NOS3*, *Nkx2.5*, and *ROBO4*) have been associated with BAV disease in patients (24).

Abnormalities in the amount of FBN-1 have been found in the aorta of patients with BAV and patients undergoing aortic surgery (37). Although FBN-1 has received much more attention than FBN-2 in the context of aortopathy, there is evidence for the involvement of the latter in aneurysm development and BAV disease. It has been recently shown that patients with BAV have increased expression levels of *FBN-2* in the ascending aorta (5). In our hamster model, we found a significant elevation of the *Fbn-2* expression levels, leading to an abnormal ratio of *Fbn1*/*Fbn-2* expression. It is possible that this *Fbn* isoform expression shift in the aorta alters the architecture of the microfibril causing matrix degeneration, SMC detachment and apoptosis. Although both FBN-1 and FBN-2 form part of the microfibril, the former is the main adult isoform whereas the latter is normally expressed during embryonic development, and both have partially overlapped functions in vascular matrix microfibril formation (38). However, FBN-2 may be a more relevant FBN isoform in the embryonic development of BAV, as this isoform is involved in the process of endocardial-to-mesenchymal transformation (39, 40), which is responsible for the formation of the aortic valve primordia and has been shown to be a key BAV development mechanism (5, 19). Consequently, our results support the hypothesis that defective *Fbn* expression, particularly *Fbn-2* may be an early trigger for the development of both a BAV and a structurally abnormal aorta (3, 5).

Although fragmentation of elastic lamellae is a hallmark of cystic medial degeneration causing BAV aortopathy, we did not find signs of elastic fragmentation in hamsters. However, we observed disorganization of the normally parallel elastic sheets. These were more undulated in animals of the T strain, what has been confirmed by quantification of the waviness index. Interestingly, a significantly higher waviness index has been detected in the aorta of patients with Ehlers-Danlos type IV syndrome, irrespective of vessel size or age (41).

TGF-β is a key molecule in the regulation of *Fbn* expression and in homeostasis maintenance in the vascular extracellular matrix (42). A common feature of syndromic aneurysms, including BAV disease appears to be dysregulation of TGF-β signaling (6, 7). In the hamster model, the expression levels of *Tgf*-β were significantly increased in the aortic wall, irrespective of aortic valve morphology. These data support the hypothesis that TGF-β signaling through FBN is an early trigger of the aortic wall alterations.

Endothelial nitric oxide synthase deregulation in the aortic wall is believed to be central in aneurysm formation (9, 10). Patients with AD show varying expression levels of the eNOS protein in different areas (proximal, distal, convexity, and concavity) of the aneurysmal aorta (9). *eNOS* is known to be upregulated by elevated fluid shear stress (8), but it is unclear to what extent the increased expression of *eNOS* in the aortic wall of BAV patients is due to hemodynamic alterations caused by the bicuspid morphology of the aortic valve (10). Our results in the hamster model indicate that eNOS protein expression is significantly reduced in the aortic media, especially in the convexity of animals of the T strain, irrespective of the aortic valve morphology. This suggests that eNOS dysregulation is an intrinsic characteristic of the BAV-associated aortic wall defect, and not a consequence of altered hemodynamics. Recently, Gauer et al. (43) found that dysregulated eNOS occurs independent of dilation in human BAV aortas and suggested that these changes are the result of an underlying genetic factor, rather than altered hemodynamics.

Multiple studies have shown increased MMP activity in the media of aneurysmatic aortas, suggesting a central role of these proteases in the degradation of the extracellular matrix leading to aortopathy (37, 44, 45). While MMP-9 seems to be linked to aneurism development in the abdominal aorta associated with atherosclerotic lesions, MMP-2 is specifically upregulated in the ascending aorta of patients with BAV (44, 45). Our results in the aorta of hamsters show that *Mmp-9* expression increases with age, although differences were only significant for T-TAV animals, but do not significantly change according to the strain or the aortic valve morphology of the animals. Nevertheless, *Mmp-2* expression is significantly reduced in the aorta of animals with BAV, regardless of age. This opposite change in *Mmp-2* expression compared to humans may indicate the existence of species-specific differences in the homeostasis of the vascular extracellular matrix. It may well be that MMP-2 reduction in the aorta of hamsters with BAV acts as a protective factor, being responsible for the lack of progression to aortopathy in this model.

Currently, it is still debated whether the etiological link between BAV and AD in patients is the result of an altered hemodynamic or an underlying genetic defect, most research favoring the combination of both factors as the main trigger (6, 11–15). Although different lines of evidence support the genetic hypothesis, none of them serve as a proof of concept (6, 11). In the present study, both morphological and molecular, quantitative and qualitative differences, for all parameters studied, were obtained when comparing animals from two strains with different genetic backgrounds, but not when comparing genetically uniform animals with TAV and BAV from the T strain. These results indicate that in this model, a strain specific genotype causes a structurally abnormal ascending aortic wall together with a low penetrant BAV phenotype, pointing to a genetic causality and excluding the participation of hemodynamic factors. Thus, the results of the present study constitute direct experimental evidence demonstrating a genetic link between structural abnormalities of the aortic valve and ascending aorta in a spontaneous animal model.

Previous work showed that BAV in hamsters result from defective behavior of the neural crest cells that colonize the embryonic cardiac outflow tract (19, 26, 28). These cells migrate from the embryonic circumpharyngeal region to form the walls of the aortic arch arteries and part of the intrapericardial arterial trunks and semilunar valves (40, 46–48). According to our present results, abnormal neural crest cells in hamster embryos may cause a structural defect of the ascending aorta, together with a low penetrant BAV phenotype. Incomplete penetrance of the BAV phenotype occurs not only in the hamster model (25–29), but also in most mutant mouse models (28), as well as in humans (11, 31, 32). The penetrance of a trait can be reduced by allelic variability and/or by the influence of epigenetic and environmental factors such as intangible variation or developmental noise, the latter defined as stochastic fluctuations in gene expression resulting in phenotypic changes even when genetic or environmental factors are fixed (25, 27, 29, 49). Moreover, the same phenotypic variation has been described in twin patients, one of whom develops a BAV while the other shows a normal TAV morphotype (30). Whatever the origin of the incomplete BAV penetrance is, when we extrapolate all these data to the clinical context, it can be deduced that a portion of the patients with AD and a normal TAV are indeed affected by bicuspid aortopathy.

This extrapolation may help explain why knowledge on BAV aortopathy pathogenesis remains elusive, as recently alerted by specialists (24). Early studies on BAV disease showed that pathogenesis of AD in patients with TAV and BAV is similar, although the latter manifest a more severe disease at a younger age. However, later studies revealed qualitative differences in the protein expression profile of diseased aortas from TAV and BAV patients, suggesting different etiologies (24, 50). The experimental strategies used in these studies are usually based on comparisons between samples from TAV and BAV patients with or without aortopathy. This strategy however is not able to distinguish between TAV patients with a distinct aortopathy etiology and TAV patients with an aortopathy etiology similar to that of BAV patients. Our present results show that this patient population (i.e., BAV aortopathy in TAV patients) indeed probably exists. Some patients with a TAV may have a structurally abnormal aorta resulting from a BAV-associated aortopathy predisposing genotype, showing the same risk to develop AD as a BAV patient. Indeed, studies with human pedigrees already favor this hypothesis, as first-degree relatives of BAV patients owing a normal TAV show a higher risk of AD (51–53). Therefore, aortic valve anatomy cannot be considered a precise predictive and diagnostic biomarker for BAV aortopathy, because the genetic defect conferring disease predisposition does not have to concordantly affect aortic valve morphology and ascending aorta structure. This notion should also be considered when establishing patient experimental groups in studies looking for disease biomarkers and mechanistic approaches, because a TAV patient sample could be incorrectly included in a control group, while it would really correspond to a highly predisposed patient.

## Conclusion

In summary, the results of the present study indicate that the hamster model of BAV includes not only a defective valve, but a structural abnormal aortic wall as well. Thus, we present the first spontaneous animal model of BAV aortopathy predisposition. Given that aortic wall alterations were equally present in animals with TAV and BAV of the affected isogenic strain, our results constitute direct experimental evidence supporting the genetic hypothesis on the etiology of BAV aortopathy. This model may represent a group of patients in which abnormal migration of embryonic neural crest cells during cardiac outflow tract development leads to a structural defect of the ascending aortic media, together with a low penetrant BAV. The aortic media defect may be caused by abnormal expression of *Tgf*-β and *Fbn-2* that alters the basic structure of the elastic lamellae, causing age-associated SMC apoptosis mediated by nitric oxide downregulation. In addition, these results allow raising the hypothesis that a significant human population with normal, tricuspid valves is in risk to aortopathy, similar to a population of BAV patients with whom they share the same genetic predisposition. This should be taken in consideration for clinical management of aortopathy progression, and for the establishment of experimental groups in research dealing with biomarker and pathophysiological mechanism discovery.

## Data availability statement

The original contributions presented in the study are included in the article/Supplementary material, further inquiries can be directed to the corresponding author.

## Ethics statement

The animal study was reviewed and approved by the Ethics Committee of Animal Experiments of the University of Málaga (CEUMA; Ethics authorization number: 25/08/2021/118).

## Author contributions

BF and AD: conception of the work. BF and FP-M: design of the work. MS-N, BP-V, ML-U, CR-M, and MF: acquisition and analysis of data for the work. MS-N, BP-V, ML-U, CR-M, MF, AD, FP-M, JR-C, and BF: interpretation of data for the work and revising the work. BF: drafting the work. All authors approved the final version to be published and agreed to be accountable for all aspects of the work.
