# Supplementary material for: Experimental evidence of the genetic hypothesis on the etiology of bicuspid aortic valve aortopathy in the hamster model
Source: Front Cardiovasc Med. 2022 Aug 8;9:928362. doi: 10.3389/fcvm.2022.928362 (PMC9393263; doi:10.3389/fcvm.2022.928362)
Supplement: Supplementary file 11 [file Table_1.docx]

**Experimental evidence of the genetic hypothesis on the etiology of bicuspid aortic valve aortopathy in the hamster model**

**Etiology of bicuspid aortic valve aortopathy**

María Teresa Soto-Navarrete^a,b^, Bárbara Pozo-Vilumbrales^a,b^, Miguel Ángel López-Unzu^a,b^, Carmen Rueda-Martínez^a^, María Carmen Fernández^a,b^, Ana Carmen Durán^a,b^, Francisco Javier Pavón^b,c,d^, Jorge Rodríguez-Capitán^b,c,d^, Borja Fernández^a,b,d^

**Supplemental Appendix**

**Supplement Figure 1.** Autofluorescence of elastic lamina (green) in a histological section of a TTAV animal used to calculate the waviness index. The length of an elastic lamella (handmade white line; L_h_) and the straight line connecting the ends of the lamella (yellow line; L_0_) are labelled (waviness index=L_0_/L_h_). Scale bar: 100 μm

**Supplement Table 1.** Primer sequences for the target genes.

| **Gen Symbol** | **Foward sequences** | **Reverse sequences** | **Accession** |
| --- | --- | --- | --- |
| ***Tgf-β*** | GGCTACCACGCCAACTTCTG | GAGGGCAAGGACCTTACTGTACTG | Ref. 1 |
| ***Fbn-1*** | AGCACAAACGAAACAGACGC | TTCTCCACATCCCAACTCGC | XM_005068561.1 |
| ***Fbn-2*** | AGCCAGCCTTCAGAAGTGTTT | CCCTTCTGTGAGGGTCAACAT | XM_005069091.1 |
| ***Mmp-2*** | TTGATGGCATCGCTCAGATC | CTGCGAAGAACACAGCCTTC | Ref. 2 |
| ***Mmp-9*** | CACTCGCTTGGATAGGATGTT | TTGGTCGTGGCAGAAATAGG | XM_005084984.2 |
| ***Cdkn1β*** | CAGCTTGCCGGAGTTCTACT | ATGCCGGTCCTCAGAGTTTG | XM_005072872.1 |

**Supplement Table 2.** Amplicon length in base pairs (A), correlation coefficient (R^2^) and amplification efficiency (E) of each gene studied.

| **Gen Symbol** | **A** | **R^2^** | **E (%)** |
| --- | --- | --- | --- |
| ***Tgf-β*** | 81 | 1 | 85 |
| ***Fbn-1*** | 86 | 1 | 95 |
| ***Fbn-2*** | 121 | 1 | 95 |
| ***Mmp-2*** | 186 | 1 | 85 |
| ***Mmp-9*** | 91 | 1 | 90 |
| ***Cdkn1β*** | 121 | 1 | 90 |

**Supplement Figure 2.** Analysis of the relative diameter of the ascending aorta of hamsters grouped according to the strain (A) or the valve morphology (B). BAV: bicuspid aortic valve; H: control strain; T: affected strain; TAV: tricuspid aortic valve (from H and T strains). Only significant p-values are detailed.

**Supplement Figure 3.** Quantification of the waviness index in the aortic convexity and concavity of animals grouped according to the strain (A) or the valve morphology (B). BAV: bicuspid aortic valve; H: control strain; T: affected strain; TAV: tricuspid aortic valve (from H and T strains). Only significant p-values are detailed.

**Supplement Figure 4.** Quantification of the percentage of TUNEL^+^ cells revealed significant differences between convexity and concavity, and between the affected (T) and control (H) strains for each segment (A). No significant difference was obtained when comparing BAV and TAV animals from both strains (B). Only significant p-values are detailed.

**Supplement Figure 5.** Quantification of the aortic media area occupied by eNOS^+^ cells. Expression was reduced almost by halve in the convexity and by one third in the concavity of animals of the T strain (A). When animals were grouped according to the aortic valve morphology, a similar reduction was observed in animals with BAV compared with animals with TAV (B). BAV: bicuspid aortic valve; H: control strain; T: affected strain; TAV: tricuspid aortic valve. Only significant p-values are detailed.

**Supplement Figure 6.** Analysis of *Tgf-β* (A), *Fbn-1* (B), *Fbn-2* (C) and *Mmp-2* (D) mRNA expression in the ascending aorta of adult (grey bars) and old (solid bars) hamsters grouped according to the strain and the valve morphology. The expression was normalized against the reference gene *Cdkn1β*. H: control strain; TBAV: bicuspid aortic valve from the affected strain; TTAV: tricuspid aortic valve from the affected strain.

**Supplement Figure 7.** Analysis of *Tgf-β* mRNA expression in the ascending aorta of hamsters grouped according to the strain (A) or the valve morphology (B). *Tgf-β* expression significantly increased in the affected strain. The expression was normalized against the reference gene *Cdkn1β*. H: control strain; BAV: bicuspid aortic valve; T: affected strain; TAV: tricuspid aortic valve.

**Supplement Figure 8.** Analysis of *Mmp-2* mRNA expression in the ascending aorta of hamsters grouped according to the strain (A) or the valve morphology (B). *Mmp-2* expression was significantly reduced in animals with BAV. The expression was normalized against the reference gene *Cdkn1β*. H: control strain; BAV: bicuspid aortic valve; T: affected strain; TAV: tricuspid aortic valve.

**Supplement Figure 9.** Analysis of *Mmp-9* mRNA expression in the ascending aorta of adult and old hamsters grouped according to the strain (A) or the valve morphology (B). *Mmp-9* expression increases with age in all animal groups, but differences were significant only when comparing animals with TAV or animals from the T-strain. The expression was normalized against the reference gene *Cdkn1β*. H: control strain; BAV: bicuspid aortic valve; T: affected strain; TAV: tricuspid aortic valve.

**Supplement Figure 10.** Analysis of *Fbn1* (A,D), *Fbn2* (B,E) and ratio *Fbn1*/*Fbn2* (C,F) mRNA expression in the ascending aorta of hamsters grouped according to the strain (A-C) or the valve morphology (D-F). *Fbn2* increased in animals from the T-strain (B) resulting in a significant reduction of the *Fbn1/Fbn2* ratio (C). The expression was normalized against the reference gene *Cdkn1β*. H: control strain; BAV: bicuspid aortic valve; T: affected strain; TAV: tricuspid aortic valve.

**References**

1. Espitia CM, Zhao W, Saldarriaga O, et al. Duplex real-time reverse transcriptase PCR to determine cytokine mRNA expression in a hamster model of New World cutaneous leishmaniasis. *BMC Immunol* 2010:**11**(1):1-12.

2. Prakobwong S, Pinlaor S, Yongvanit P, Sithithaworn P, Pairojkul C, Hiraku Y. Time profiles of the expression of metalloproteinases, tissue inhibitors of metalloproteases, cytokines and collagens in hamsters infected with *Opisthorchis viverrini* with special reference to peribiliary fibrosis and liver injury. *Int J Parasitol* 2009;**39**(7):825-835.
